# Supplementary figures and images for: The viral packaging motor potentiates Kaposi’s sarcoma-associated herpesvirus gene expression late in infection
Source: PLoS Pathog. 2023 Apr 17;19(4):e1011163. doi: 10.1371/journal.ppat.1011163 (PMC10138851; doi:10.1371/journal.ppat.1011163)

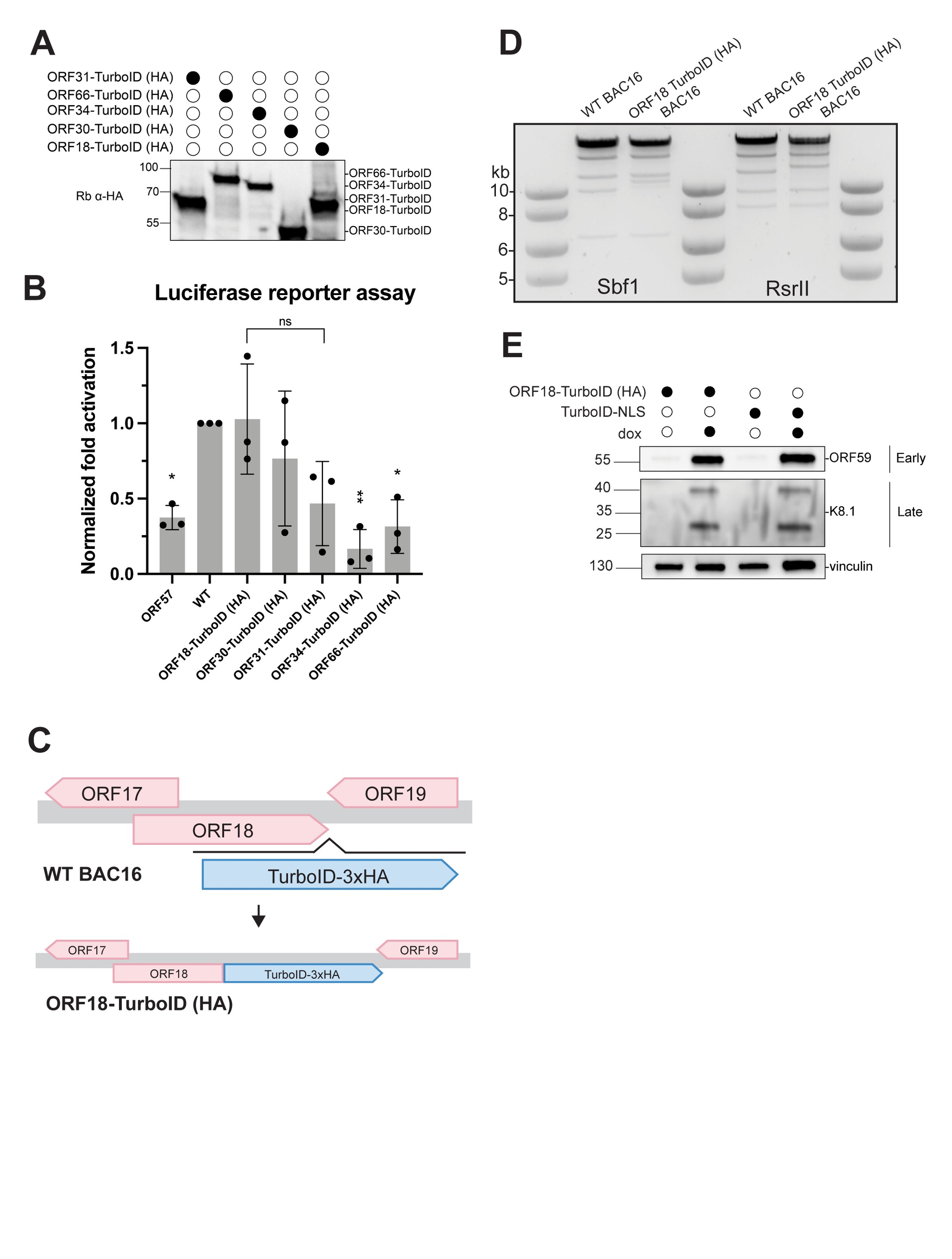

Supplement: S1 Fig — (A) Western blots of HEK293T cells transiently transfected with vTA-TurboID-3xHA constructs indicating that all vTAs tested express when fused to the ligase. (B) HEK293T cells were transiently transfected with plasmids encoding the six vTAs (either WT or TurboID-tagged as indicated), a plasmid containing the firefly luciferase gene under control of either the ORF57 or K8.1 promoter, and a plasmid encoding renilla luciferase as a transfection control. (C) Diagram showing the genomic locus of ORF18 and the site of introduction of TurboID-3xHA. ORF18 partially overlaps with ORF17 and is directly upstream of ORF19. (D) The recombinant ORF18-TurboID BAC was digested by the restriction enzymes SbfI and RsrII and compared to digested WT BAC16, which confirmed that the introduction of TurboID had not resulted in any large-scale recombination events within the BAC. (E) Western blots of iSLK lysates indicating that the addition of TurboID to ORF18 within the BAC does not impair expression of representative early or late proteins upon reactivation. (TIF) [file ppat.1011163.s004.tif]

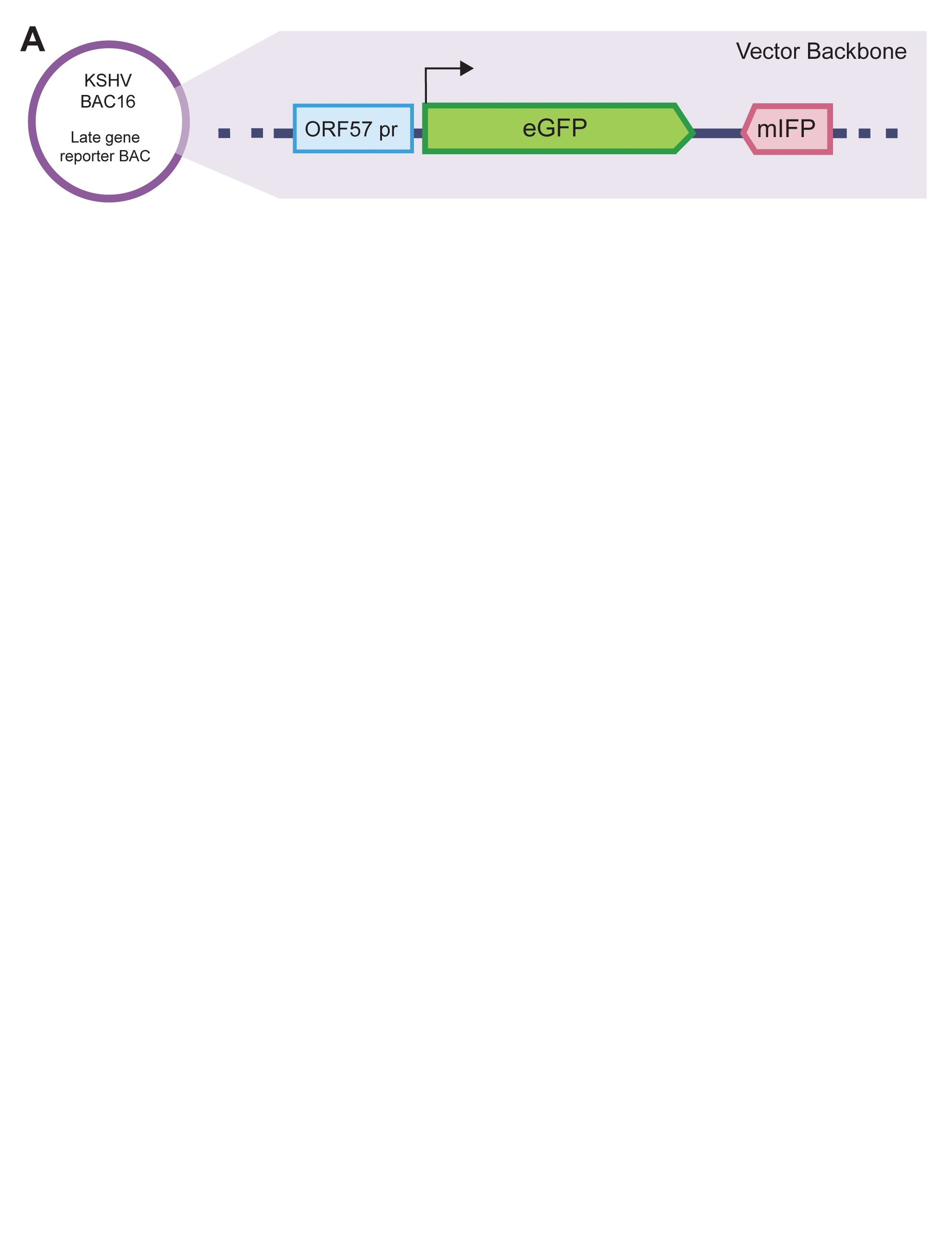

Supplement: S2 Fig — (A) Schematic of the early gene reporter virus used to measure early gene expression by flow cytometry. The reporter construct was cloned into a region of the vector backbone of the KSHV BAC16. The ORF57 early gene promoter drives expression of eGFP, and mIFP is constitutively expressed. (TIF) [file ppat.1011163.s005.tif]

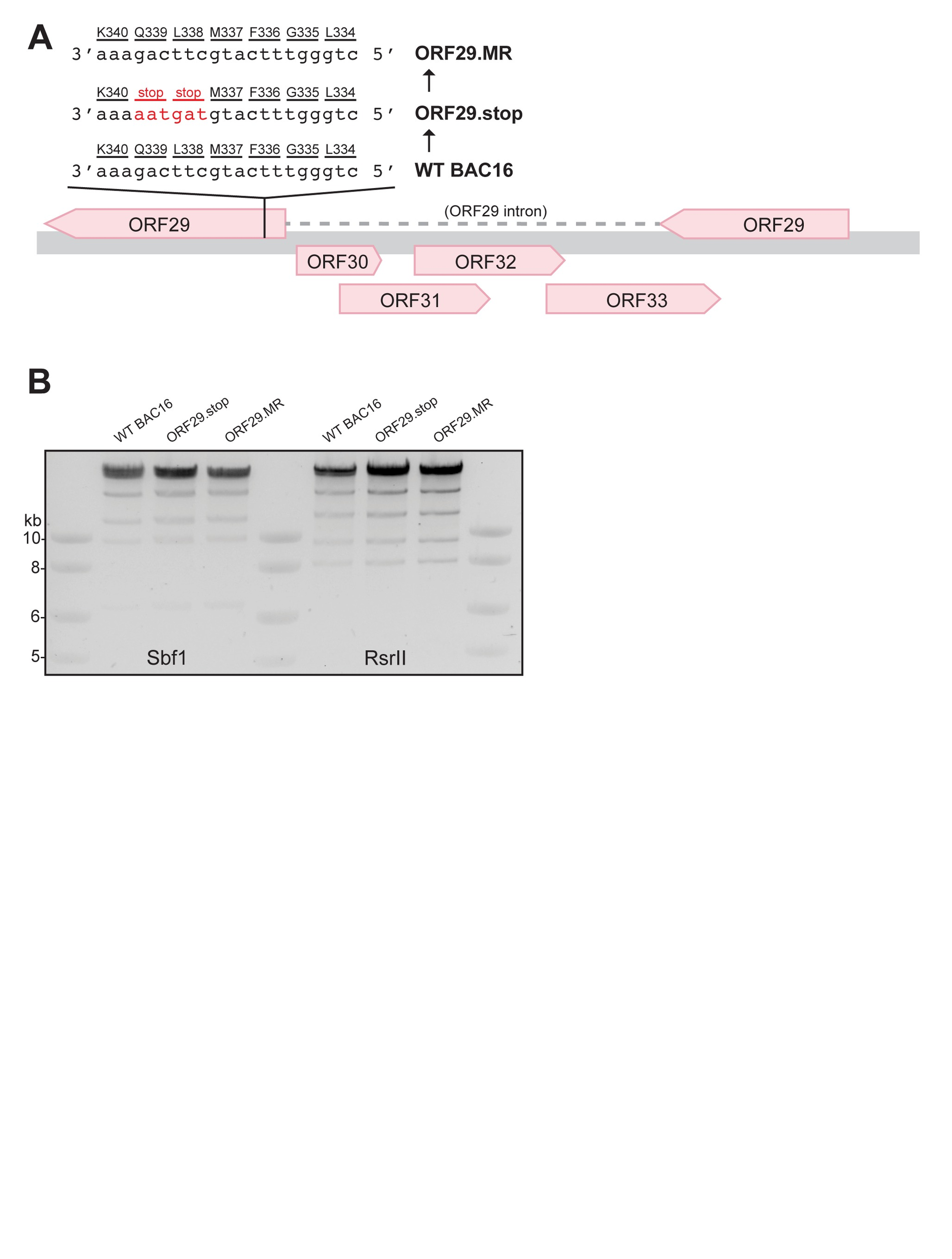

Supplement: S3 Fig — (A) Diagram showing the genomic locus of ORF29 and the site of introduction of the premature stop codons. ORF29 is composed of two exons; two premature stop codons were introduced near the start of the second exon. (B) The recombinant ORF29.stop and ORF29.MR BACs were digested by the restriction enzymes SbfI and RsrII and compared to digested WT BAC16, which confirmed that the introduction of these mutations had not resulted in any large-scale recombination events within the BAC. (TIF) [file ppat.1011163.s006.tif]

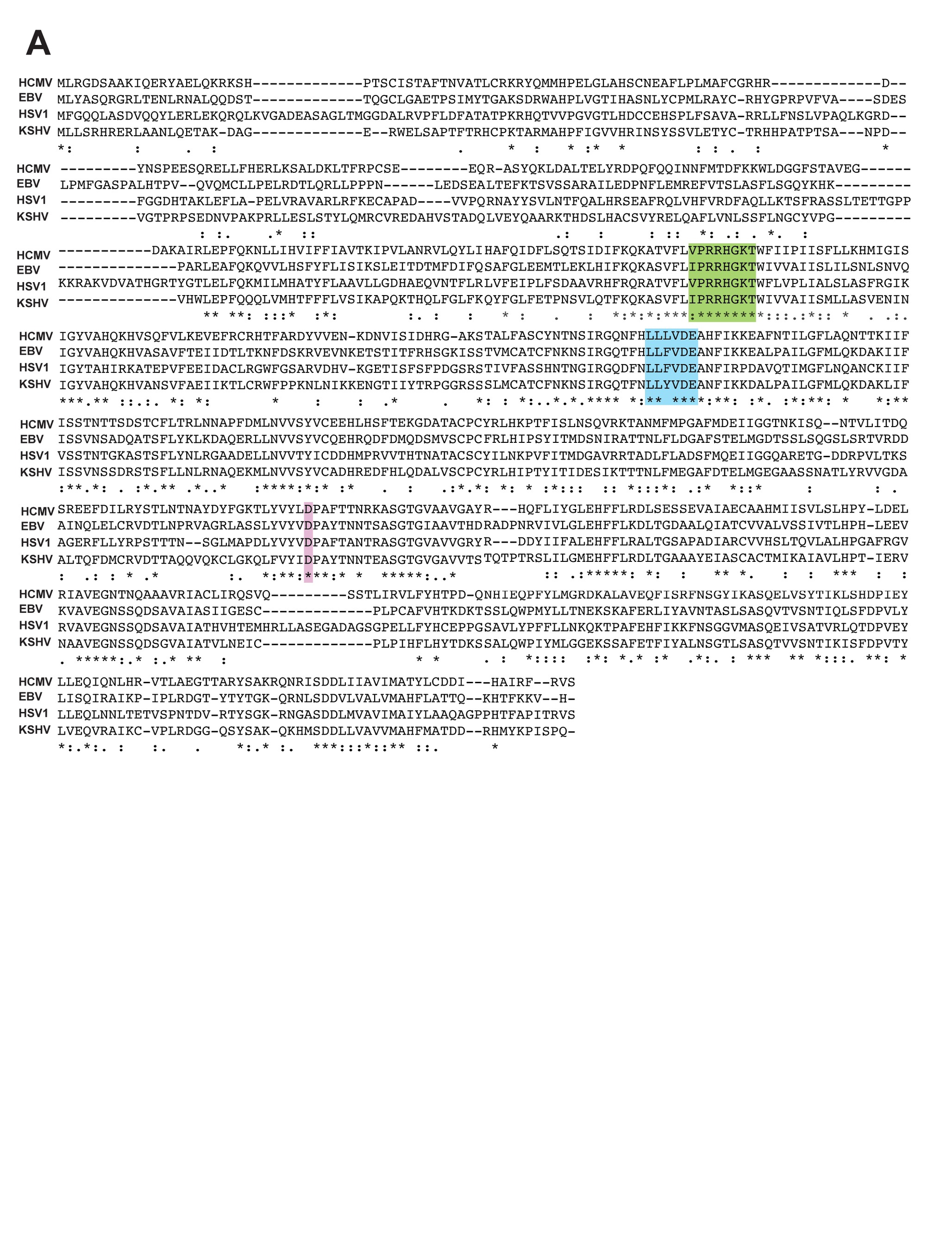

Supplement: S4 Fig — (A) Multiple sequence alignment of ORF29 and homologs from representative alpha-, beta-, and gammaherpesviruses. (*) indicates a fully conserved residue, (:) indicates conservation of residues with similar properties, (.) indicates conservation of residues with weakly similar properties. The Walker A and Walker B motifs are highlighted in green and blue respectively. The aspartic acid required for nuclease activity at position 476 in ORF29 is highlighted in purple. Alignment generated using the T-coffee method [55]. (TIF) [file ppat.1011163.s007.tif]
