# Supplementary material for: The viral packaging motor potentiates Kaposi’s sarcoma-associated herpesvirus gene expression late in infection
Source: PLoS Pathog. 2023 Apr 17;19(4):e1011163. doi: 10.1371/journal.ppat.1011163 (PMC10138851; doi:10.1371/journal.ppat.1011163)
Supplement: Attachment — Submitted filename: Reviewer responses and detailed changes.docx [file ppat.1011163.s008.docx]

**Responses to all review comments:**

Major Issues:

Reviewer #3: Only minor comments would be to repeat the western blots on a longer time course for early gene products in the KO and MR strains to clarify whether the expression defect of early genes is consistent or discordant between RNA and protein. What might the potential mechanism for early gene expression defects be at the 48h time point?

Response: While optimizing conditions for the CRISPR screen, we observed by flow cytometry that while late gene reporter GFP signal was detectable by 48 h post reactivation in WT cells, the population was very heterogeneous (likely due to asynchronous reactivation). By 72 h, the population was much more homogenous. We reported protein expression data at 72 h post reactivation for WT and the ORF29.stop virus because we believe this time point best represents the total population of cells. Additionally, ORF29 protein does not appear until 36 h post reactivation, and our data suggests perturbations to viral mRNA levels in the ORF29.stop virus only manifest after that. Given that changes in mRNA levels take additional time to become evident as changes at the protein level, we believe 72 h post reactivation is the most representative time to observe both early and late protein levels for the ORF29.stop and MR cell lines.

Minor issues:

Reviewer #1: 1) Lines 104-110. The assay described here (Fig S1B), unlike the excellent one used in Figure 2, should not be referred to as a "late" gene reporter assay. It is well established that authentic late gene transcription occurs in viral replication compartments (PMID: 23552415), even in alphaherpesviruses (PMID: 21555562) and requires continuous viral DNA replication (PMID: 29813138). The HEK293 cells used in this assay (as described in lines 652-663) harbor neither KSHV nor viral replication compartments and therefore cannot authentically report late gene transcription. I accept that, for reasons that are not entirely clear, this assay can distinguish between functional and non-functional vTA fusion proteins - probably via a transcription mechanisms that more closely approximates chromatinized transcription. Moreover, this assay is not required to established that ORF18-TurboID works as this is unequivocally established by Figs S1E and Fig1B. The risk of legitimizing this "late" reporter assay is that less sophisticated investigators could then use it to show that late gene transcription can occur in the absence of ORF29 (or even ORF9).

Response: We agree that the luciferase reporter assay used in Fig S1B does not capture all the regulatory features of late gene transcription, most notably the contribution of DNA replication. Nevertheless, it is a useful tool for distinguishing between functional and non-functional vTA variants. This assay correctly identified ORF18-Turbo as a viable fusion, and the assay has been validated in several of our prior publications to define functionally relevant vTA residues (Davis et al. 2016 PMID: 28336944, Castaneda 2019 PMID: 30305361, Didychuk et al. 2020 PMID: 31578296). While ORF18-TurboID function was confirmed in figures S1E and Fig 1B, this assay was a useful first step for screening potential fusion candidates. We believe including this supplementary figure is important for providing an accurate representation of the process and decision making that went into setting up the proximity labeling mass spectrometry experiment.

2) ORF31 is shown as interacting with ORF18 (Fig 1A), yet ORF31 is not shown among the KSHV proteins pulled down by ORF18-TurboID (Fig 1D) despite the claim that all of the known "ORF18-interacting proteins" were identified in the assay. Either the dotted line should be removed from Fig 1A or ORF31 added to Fig 1D.

Response: ORF31 was mistakenly not included in the original figure (1D). The figure has been updated to include ORF31 as described below.

3) I feel like the title is misleading given that ORF29 deletion impairs both early and late mRNAs. I agree with the authors that the selective effect on late proteins is likely due to translation of early mRNAs into protein prior to the ORF29 effect. However, the title implies ORF29's role in late gene transcription is on par with the vTAs.

Response: We have changed the title of the manuscript to: “The viral packaging motor potentiates KSHV gene expression late in infection.” The new title now refers to viral gene expression generally, while highlighting that ORF29’s contributions to the phenotypes we see manifest late during infection.

**Description of the changes you have made in the manuscript:**

Title: To address Reviewer #1’s concerns, we have changed the title from: “The viral packaging motor potentiates late gene expression in Kaposi's sarcoma-associated herpesvirus" to: “The viral packaging motor potentiates KSHV gene expression late in infection”.

Figure 1D: The protein ORF31 was added to the set of ORF18-interacting proteins shown in this figure that were identified by proximity labeling-mass spectrometry. This protein appeared in the dataset, but was mistakenly omitted when the figure was drawn. Additionally, the outlines of the proteins ORF30, ORF66, ORF24, ORF21, and ORF75 were changed to gold to reflect the nuclear localization of these proteins. In the original figure, these proteins lacked gold outlines due to a formatting error.

Materials and Methods: Addgene plasmid IDs have been updated.
